# OA02.01. Effect of macronutrient composition of weight loss diets on reduction of the inflammatory marker hsCRP

**DOI:** 10.1186/1472-6882-12-S1-O5

**Published:** 2012-06-12

**Authors:** J Nicklas, F Sacks, S Smith, M LeBoff, J Rood, G Bray, P Ridker

**Affiliations:** 1 Beth Israel Deaconess Medical Center, Boston, USA; 2 Department of Nutrition, Harvard School of Public Health, Boston, USA; 3 Sanford-Burnham Medical Research Institute, Winter Park, USA; 4 Brigham and Women's Hospital, Boston, USA; 5 Pennington Biomedical Research Center, Baton Rouge, USA

## Purpose

High sensitivity CRP (hsCRP), a marker of systemic inflammation, is associated with obesity and is an independent predictor for cardiovascular disease. Although practitioners may prescribe weight loss and/or special diets to treat systemic inflammation, little is known about how diets differing in fat, protein, or carbohydrate composition affect hsCRP.

## Methods

In the two-year POUNDS (Preventing Overweight Using Novel Dietary Strategies) LOST trial, overweight and obese adults were randomly allocated to one of four weight loss diets with targeted percentages of energy derived from fat/protein/carbohydrates (20/15/65%; 20/25/55%; 40/15/45%; 40/25/35%, respectively). All participants received tailored diet prescriptions with a 750 kilocalorie deficit from energy expenditure, and an intensive behavioral program accompanied all diet assignments. hsCRP as well as cardiovascular and metabolic factors were measured at baseline, 6, and 24 months among 710 participants.

## Results

There was a 25% (IQR +7%, -50%) decrease in hsCRP at 6 months in all trial participants, as well as 7% (IQR -3%, -11%) weight loss and a reduction in waist circumference by 6% (IQR -3%, -10%) (all p <.002), with no significant differences observed according to randomized dietary composition. Irrespective of diet composition, the percent change in hsCRP at 6 and 24 months correlated modestly with change in weight, waist circumference, fasting insulin, fasting glucose, HOMA (homeostasis model assessment of insulin sensitivity), and most lipid levels. Reductions in hsCRP persisted throughout the study period despite an approximate 50% regain of weight by 24 months.

## Conclusion

hsCRP decreased equally on all four weight loss diets, and was associated with improvements in lipids and metabolic factors. Findings from this study suggest that macronutrient composition is not an important component of weight loss diets designed to reduce inflammation.

